# Numerical solution for MHD peristaltic transport in an inclined nanofluid symmetric channel with porous medium

**DOI:** 10.1038/s41598-022-07193-5

**Published:** 2022-03-01

**Authors:** A. M. Abd-Alla, Esraa N. Thabet, F. S. Bayones

**Affiliations:** 1 Department of Mathematics, Faculty of Science, Sohag University, Sohag, Egypt; 2 Department of Mathematics and Statistics, College of Science, Taif University, P.O. Box 11099, Taif, 21944 Saudi Arabia

**Keywords:** Materials science, Mathematics and computing, Nanoscience and technology

## Abstract

The significance of the study is to determine of transferred heat and mass impact on the magneto-hydrodynamic peristalsis of Jeffery nanofluid through porous media with inclined symmetric channels whose walls are induced by peristaltic motion within porous media. The aim of this investagtion is to study the influence of various types of parameters such as Brownian motion, thermophoresis, buoyancy forces, and magnetic fields are studies on concentration, temperature, and axial velocity. The numerical solution has been achieved according to the long-wavelength and low Reynolds number approximation utilizing the MATLAB bvp4c function. The resultant dimensions of nonlinear governing equations were approached numerically through the Runge–Kutta- Fehlberg integration scheme, a MATLAB program. The influence of different factors such as the ratio of relaxation to retardation times, nanoparticle Grashof number, and magnetic field was discussed on concentration, temperature, and velocity profiles. tables and graphs were used to demonstrate the numerically computed numerical results. Plotting graphs were utilized for evaluating the pertinent parameters impacts on the aforementioned quantities based on computational results. According to the findings, the effect of the parameters are significant.

## Introduction

Various research associated with the peristalsis phenomenon has been adopted because of its impact in various biological and industrial methods. Furthermore, it demonstrates excellent importance because of the distinctive features of symmetric and asymmetric channel walls; contraction and compulsion. This strategy reflects another feature represented in the capability of the channel walls to move and propagate the substance across the channel walls. The significance of peristalsis can be observed in the transport processes; for instance, the urine transport via the kidneys toward the bladder, food particle movement across the digestive tract, and the motion of chyme through the gastrointestinal tract. Nevertheless, industrial applications can be observed in the lung, heart, dialysis machines, and hose-roller pumps. The modern drug delivery system relies on the peristaltic motion associated with nano-fluids. Nanoscience, the conduct of physical systems when confined to nanoscale (< 100 nm) dimensions together with the physical phenomena that take place at the nanoscale, is at this time one of the most active and quickly evolving areas of interdisciplinary research in physics, chemistry, biology, and engineering. Nanotechnology, the application of these properties and phenomena, can transform a great variety of scientific and technological fields. Some detailed applications are now available for research. The nanofluid peristaltic flow within a diverging tube was addressed by Akbar et al.^1^, where the solutions of temperature and nanoparticle equations were evaluated by the homotopy perturbation method. The MHD peristaltic transport, as well as Joule heating impacts of nanofluid in a channel with compliant walls was investigated^2^. The Joule heating and wall flexibility impacts on the conducting nanofluid peristaltic transport in a non-uniform/ uniform porous channel was evaluated^3^. The induced magnetic field impact on the mixed convection peristaltic motion of nanofluids in a vertical channel was evaluated^4^. The peristalsis numerical solution regarding Carreau–Yasuda nanofluids exposed to nonlinear thermal radiation and thermophoresis in symmetric channels with a constant magnetic field was obtained^5^. The thermal radiation impact in magnetohydrodynamic (MHD) third-grade nanofluid mixed convective peristaltic transport within a curved channel with wall properties was studied^6^. The impact of wall properties and aligned magnetic field on the peristaltic transport and heat and mass transfer of a Jeffrey/Newtonian nanofluid in a tapered channel was investigated^7^. The combined convection of Jeffrey nanofluid peristaltic flow in a channel was addressed^8^. The MHD peristalsis of Jeffrey nanomaterial in a vertical asymmetric compliant channel wall was addressed by Hayat et al.^9^. The effects of thermal radiation and Joule heating regarding the fourth-grade nano liquid MHD peristaltic motion in a channel were mentioned^10^. It was illustrated that the characteristics of the activation energy and the first-order chemical reaction considering MHD peristaltic transport of Eyring-Powell nanofluids^11^. Pandey and Chandra^12^ investigated the micropolar fluid axisymmetric flow caused by peristaltic waves by progressively dilating the amplitude. Kotnurkar and Giddaiah^13^ explored the double-diffusive convection investigation on peristaltic flow regarding the hypothesis of low Reynolds numbers and long wavelengths. Saleem et al.^14^ explained the heat transfer and peristaltic flow of phase flow (Cu/blood) and hybrid (Cu-Ag/blood), nanofluid models, within a curved tube with a quilted wall. The non-Newtonian peristaltic flow nanofluid across a non-uniform surface was investigated^15^. An analytical investigation of micropolar fluid MHD flow accross a porous medium influenced by sinusoidal peristaltic waves traveling down the channel walls was obtained by Pandey and Chaube^16^. El-Dabe et al.^17^ discovered the steady non-Newtonian nanofluid flow peristaltic motion with heat transfer accross a nonuniform inclined channel. The integrated effects of the thermophoresis, buoyancy forces, Brownian motion, and magnetic field on an incompressible Jeffrey nanofluid peristaltic flow across an asymmetric channel were investigated^18^. The inclined magnetic field impact on the peristaltic transport of a hyperbolic tangent nanofluid in an inclined channel with flexible walls was studied^19^. Also, ^20^ the combined impacts of peristalsis-and electroosmosis-driven flow regarding the nanoparticle dispersion role in electroosmosis regulated peristaltic flow of water accross a porous medium were examined. The nanofluid peristaltic transport in a channel having compliant walls was investigated^21^. The Dufour and Soret numbers impacts on non‐Newtonian micropolar fluid the peristaltic motion were discussed^22^. The electroosmosis and double-diffusive convection numerical simulation across micropolar nanofluid the peristaltic transport regarding an asymmetric microchannel was discussed^23^. The effects of convective and slip boundary conditions, hall currents, and electro-magneto-hydrodynamics on the peristaltic propulsion of nanofluids through porous symmetric microchannels was determined^24^. Jeffrey nanofluid magnetohydrodynamic peristaltic transport in an asymmetric channel was investigated ^18^. Numerical investigations of the dusty nanofluids peristaltic motion within a curved channel were performed^25^. The endoscope influence and heat transfer on Jeffrey fluid peristaltic flow via the gap between concentric uniform tubes was studied^26^. The impacts of fractional Maxwell fluids and Magnetic on peristaltic flows in a circular cylinder tube having heat and mass transfer were studied^27^. Recently, scientists have considered the flow of non-Newtonian and Newtonian fluids^28–47^.

This research is intended to determine the impacts of the gravitational forces, buoyancy forces, and magnetic field on velocity profiles, temperature, and concentration through porous media. First, the relevant equations for the fluid are modeled, and then the resulting mathematical problem is solved under a long-wavelength and low Reynolds number approximation. The Runge–Kutta scheme was utilized to numerically approach, the resulting dimensions of nonlinear governing equations. The physical characteristics of emerging factors were considered by drawing concentration, velocity, and temperature graphs. The findings and discussions demonstrated in the current article can be valuable for understanding Jeffrey nanofluids MHD peristaltic motion within an inclined symmetric channel. Graphs showing the concentration, temperature, and axial velocity for several parameter values have been presented and discussed.

## Formulation of the problem

**Figure 1:**
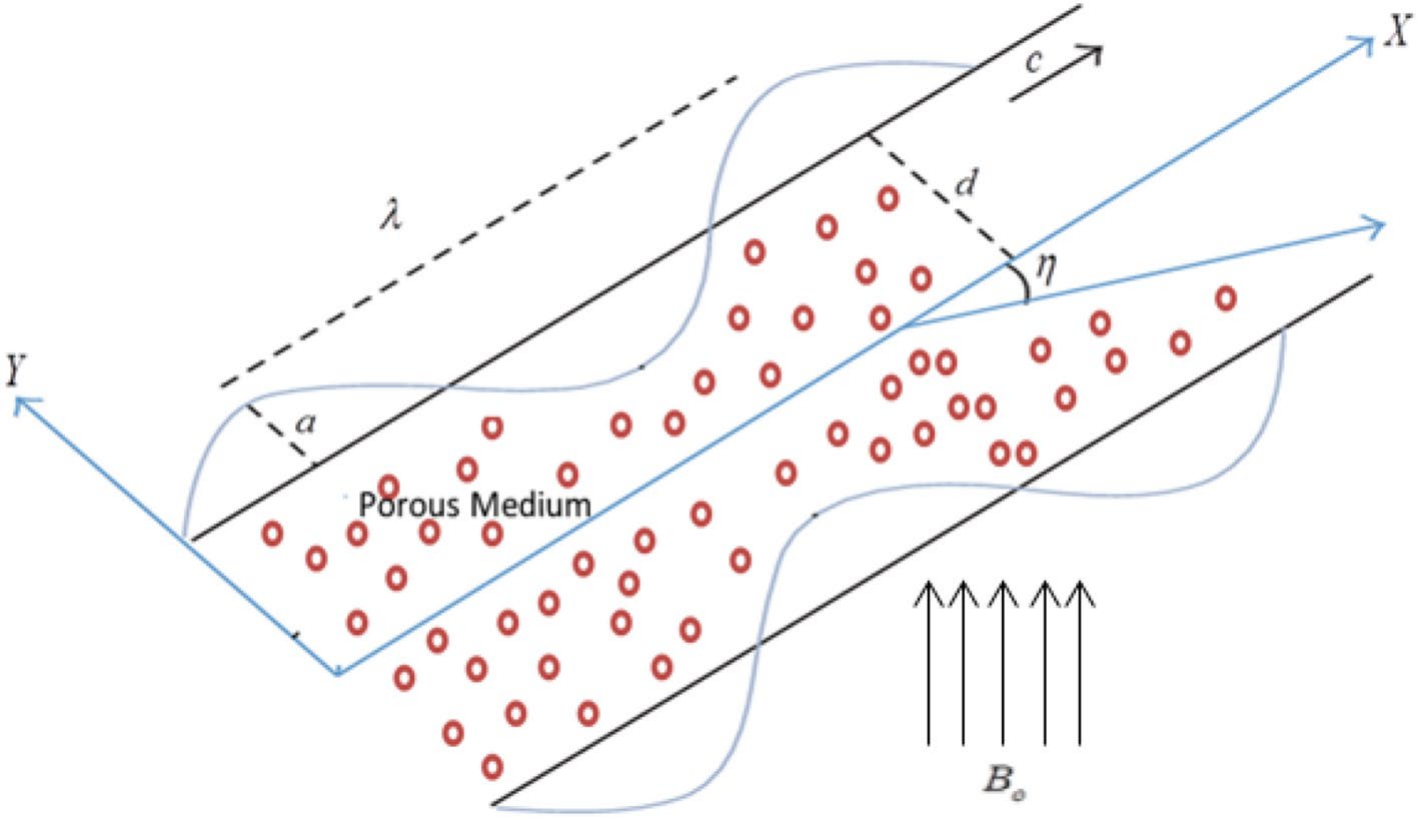
Geometrical elucidation of the physical problem.

Consider the movement of Jeffrey electrically conducting nanofluid incompressible peristaltic flow within a two-dimensional inclined symmetric porous channel. Peristaltic flow is induced by traveling sinusoidal waves advancing with constant velocity $c$ toward $(X - axis)$, a uniform magnetic field of strength $B_{0}$ is imposed normal to the channel walls $(Y - axis).$ The flow coordinate system and physical diagram are demonstrated in Fig. 1. Thermophoresis effects and Brownian motion are retained. The elimination of the induced magnetic field was due to the small magnetic Reynolds number. The following equation describes the geometry of the two wall surfaces:

1 $$\overline{H} = \pm \left[ {d + a\sin (\frac{2\pi }{\lambda }(\overline{X} - c\overline{t} ))} \right].$$

The Cauchy stress tensor and extra stress tensor ${\rm T}$ and $S$ regarding an incompressible Jeffrey material are expressed by the following equation:

2 $$\begin{gathered} {\rm T} = - PI + S,\, \hfill \\ \,S = \frac{\mu }{{1 + \lambda_{1} }}\left( {\mathop \gamma \limits^{.} + \lambda_{2} \frac{{d\mathop \gamma \limits^{.} }}{dt}} \right) \hfill \\ \end{gathered}$$

where, the pressure and the identity tensor are represented by p and $I$, respectively.

The incompressible nanofluid flow in the fixed frame are represented by:

3 $$\frac{{\partial \overline{U} }}{{\partial \overline{X} }} + \frac{{\partial \overline{V} }}{{\partial \overline{Y} }} = 0,$$

4 $$\begin{gathered} \rho_{f} \left[ {\frac{{\partial \overline{U} }}{{\partial \overline{t} }} + \overline{U} \frac{{\partial \overline{U} }}{{\partial \overline{X} }} + \overline{V} \frac{{\partial \overline{U} }}{{\partial \overline{Y} }}} \right] = - \frac{{\partial \overline{P} }}{{\partial \overline{X} }} + \frac{\partial }{{\partial \overline{X} }}\left( {\overline{S}_{{\overline{X} \overline{X} }} } \right) + \frac{\partial }{{\partial \overline{Y} }}\left( {\overline{S}_{{\overline{X} \overline{Y} }} } \right) + (1 - C_{o} )\rho_{f} g\beta_{t} \left( {\overline{T} - T_{o} } \right) \hfill \\ \,\,\,\,\,\,\,\,\,\,\,\,\,\,\,\,\,\,\,\,\,\,\,\,\, + \left( {\frac{{\rho_{p} - \rho_{f} }}{{\rho_{f} }}} \right)g\beta_{c} \left( {\overline{C} - C_{o} } \right) - \sigma B_{o}^{2} \overline{U} - \frac{\mu }{{k_{1} }}\overline{U} + \rho_{f} g\sin \eta , \hfill \\ \end{gathered}$$

5 $$\rho_{f} \left[ {\frac{{\partial \overline{V} }}{{\partial \overline{t} }} + \overline{U} \frac{{\partial \overline{V} }}{{\partial \overline{X} }} + \overline{V} \frac{{\partial \overline{V} }}{{\partial \overline{Y} }}} \right] = - \frac{{\partial \overline{P} }}{{\partial \overline{Y} }} + \frac{\partial }{{\partial \overline{X} }}\left( {\overline{S}_{{\overline{X} \overline{Y} }} } \right) + \frac{\partial }{{\partial \overline{Y} }}\left( {\overline{S}_{{\overline{Y} \overline{Y} }} } \right) - \frac{\mu }{{k_{1} }}\overline{V} - \rho_{f} g\cos \eta ,$$

6 $$\begin{gathered} \left[ {\frac{{\partial \overline{T} }}{{\partial \overline{t} }} + \overline{U} \frac{{\partial \overline{T} }}{{\partial \overline{X} }} + \overline{V} \frac{{\partial \overline{T} }}{{\partial \overline{Y} }}} \right] = \alpha \left( {\frac{{\partial^{2} \overline{T} }}{{\partial \overline{X}^{2} }} + \frac{{\partial^{2} \overline{T} }}{{\partial \overline{Y}^{2} }}} \right) + \frac{1}{{\rho_{f} c_{f} }}\left[ {\overline{S}_{{\overline{X} \overline{X} }} \frac{{\partial \overline{U} }}{{\partial \overline{X} }} + \overline{S}_{{\overline{X} \overline{Y} }} \left( {\frac{{\partial \overline{U} }}{{\partial \overline{Y} }} + \frac{{\partial \overline{V} }}{{\partial \overline{X} }}} \right) + \overline{S}_{{\overline{Y} \overline{Y} }} \frac{{\partial \overline{V} }}{{\partial \overline{X} }}} \right] \hfill \\ \,\,\,\,\,\,\,\,\,\,\,\,\,\,\,\,\,\,\,\,\,\,\,\,\, + \tau \left[ {D_{B} \left( {\frac{{\partial \overline{C} }}{{\partial \overline{X} }}\frac{{\partial \overline{T} }}{{\partial \overline{X} }} + \frac{{\partial \overline{C} }}{{\partial \overline{Y} }}\frac{{\partial \overline{T} }}{{\partial \overline{Y} }}} \right) + \frac{{D_{T} }}{{T_{m} }}\left( {\left( {\frac{{\partial \overline{T} }}{{\partial \overline{X} }}} \right)^{2} + \left( {\frac{{\partial \overline{T} }}{{\partial \overline{Y} }}} \right)^{2} } \right)} \right] + \frac{{\sigma B_{o}^{2} \overline{U}^{2} }}{{\rho_{f} c_{f} }}, \hfill \\ \end{gathered}$$

7 $$\left[ {\frac{{\partial \overline{C} }}{{\partial \overline{t} }} + \overline{U} \frac{{\partial \overline{C} }}{{\partial \overline{X} }} + \overline{V} \frac{{\partial \overline{C} }}{{\partial \overline{Y} }}} \right] = D_{B} \left( {\frac{{\partial^{2} \overline{C} }}{{\partial \overline{X}^{2} }} + \frac{{\partial^{2} \overline{C} }}{{\partial \overline{Y}^{2} }}} \right) + \frac{{D_{T} }}{{T_{m} }}\left( {\frac{{\partial^{2} \overline{T} }}{{\partial \overline{X}^{2} }} + \frac{{\partial^{2} \overline{T} }}{{\partial \overline{Y}^{2} }}} \right).$$

The flow is believed to be steady in the wave frame $(\overline{x} ,\overline{y} )$ moving with velocity $c$ from the fixed frame $(\overline{X} ,\overline{Y} ).$ The transformations between the laboratory and wave frame are given by:

8 $$\overline{x} = \overline{X} + c\overline{t} , \quad \overline{y} = \overline{Y} , \quad \overline{u} = \overline{U} - c, \quad \overline{v} = \overline{V} , \quad \overline{p} (\overline{x} ,\overline{y} ) = \overline{P} (\overline{X} ,\overline{Y} ,\overline{t} ),\quad T = \overline{T} .$$

Now, impose the non-dimension parameters and variables;

$$x = \frac{{\overline{x} }}{\lambda },$$ $$y = \frac{{\overline{y} }}{d},$$ $$u = \frac{{\overline{u} }}{c},$$ $$v = \frac{{\overline{v} }}{c},$$ $$t = \frac{{c\overline{t} }}{\lambda },$$ $$h = \frac{{\overline{H} }}{d},$$ $$\delta = \frac{d}{\lambda },$$ $$p = \frac{{d^{2} \overline{p} }}{c\lambda \mu },$$

$$M = \sqrt {\frac{\sigma }{\mu }} B_{0} d,$$ $$\overline{{\lambda_{2} }} = \frac{{\lambda_{2} c}}{d},$$ $$\Pr = \frac{\upsilon }{\alpha },$$ $${\text{Re}} = \frac{cd}{\upsilon },$$ $$S = \frac{{\overline{S} d}}{\mu c},$$ $$Fr = \frac{{c^{2} }}{gd},$$

$$Gm = \frac{{\left( {\rho_{c} - \rho_{f} } \right)g\beta_{c} \left( {C_{1} - C_{o} } \right)d^{2} }}{\mu c},$$$$Nt = \frac{{\tau D_{T} (T_{1} - T_{o} )}}{{\upsilon T_{o} }},$$$$Nb = \frac{{\tau D_{B} (C_{1} - C_{o} )}}{\upsilon },$$$$Gr = \frac{{\left( {1 - C_{o} } \right)\rho_{f} g\beta_{t} \left( {T_{1} - T_{o} } \right)d^{2} }}{\mu c},$$$$Ec = \frac{{c^{2} }}{{c_{f} \left( {T_{1} - T_{0} } \right)}},$$$$Da = \frac{{k_{1} }}{{d^{2} }},$$$$Br = \Pr \times Ec,$$

9 $$\theta = \frac{{\overline{T} - T_{0} }}{{T_{1} - T_{0} }}, \quad \Theta = \frac{{\overline{C} - C_{0} }}{{C_{1} - C_{0} }}, \quad \varepsilon = \frac{a}{d}.$$

## Solution to problem

Employing the non-dimensional variables abovementioned modifications, the previous equations can be represented by:

10 $$\begin{gathered} {\text{Re}} \delta \left[ {\left( {u + 1} \right)\frac{\partial u}{{\partial x}} + \frac{v}{\delta }\frac{\partial u}{{\partial y}}} \right] = - \frac{dp}{{dx}} + \delta \frac{\partial }{\partial x}\left( {S_{xx} } \right) + \frac{\partial }{\partial y}\left( {S_{xy} } \right) + Gr\theta + Gm\Theta \hfill \\ \,\,\,\,\,\,\,\,\,\,\,\,\,\,\,\,\,\,\,\,\,\,\,\,\,\,\,\,\,\,\,\,\,\,\,\,\,\,\,\, - \left( {M^{2} + \frac{1}{Da}} \right) \times \left( {u + 1} \right) + \frac{{\text{Re}}}{Fr}\sin \eta , \hfill \\ \end{gathered}$$

11 $${\text{Re}} \delta^{3} \left[ {\left( {u + 1} \right)\frac{\partial v}{{\partial x}} + \frac{v}{\delta }\frac{\partial v}{{\partial y}}} \right] = - \frac{\partial p}{{\partial y}} + \delta^{2} \frac{\partial }{\partial x}\left( {S_{xy} } \right) + \delta \frac{\partial }{\partial y}\left( {S_{yy} } \right) - \frac{\delta }{Da}v + \delta \frac{{\text{Re}}}{Fr}\cos \eta ,$$

12 $$\begin{gathered} {\text{Re}} \delta \left[ {\left( {u + 1} \right)\frac{\partial \theta }{{\partial x}} + \frac{v}{\delta }\frac{\partial \theta }{{\partial y}}} \right] = Ec\left[ {\delta \,S_{xx} \frac{\partial u}{{\partial x}} + S_{xx} \left( {\frac{\partial u}{{\partial y}} + \delta^{2} \frac{\partial v}{{\partial x}}} \right) + \,S_{yy} \frac{\partial v}{{\partial y}} + M^{2} \left( {u + 1} \right)^{2} } \right] \hfill \\ \,\,\,\,\,\,\,\,\,\,\,\,\,\,\,\,\,\,\,\,\,\,\,\,\,\, + \frac{1}{\Pr }\left[ {\delta^{2} \frac{{\partial^{2} \theta }}{{\partial x^{2} }} + \frac{{\partial^{2} \theta }}{{\partial y^{2} }}} \right] + Nb\left[ {\delta^{2} \frac{\partial \theta }{{\partial x}}\frac{\partial \Theta }{{\partial x}} + \frac{\partial \theta }{{\partial y}}\frac{\partial \Theta }{{\partial y}}} \right] + Nt\left[ {\delta^{2} \left( {\frac{\partial \theta }{{\partial x}}} \right)^{2} + \left( {\frac{\partial \theta }{{\partial y}}} \right)^{2} } \right], \hfill \\ \end{gathered}$$

13 $${\text{Re}} \delta \left[ {\left( {u + 1} \right)\frac{\partial \Theta }{{\partial x}} + \frac{v}{\delta }\frac{\partial \Theta }{{\partial y}}} \right] = \left( {\delta^{2} \frac{{\partial^{2} \Theta }}{{\partial x^{2} }} + \frac{{\partial^{2} \Theta }}{{\partial y^{2} }}} \right) + \frac{Nt}{{Nb}}\left( {\delta^{2} \frac{{\partial^{2} \theta }}{{\partial x^{2} }} + \frac{{\partial^{2} \theta }}{{\partial y^{2} }}} \right),$$

Now inserting the stream function $\psi$ and velocity fields designated though

14 $$u = \frac{\partial \psi }{{\partial y}},\,\,\,\,\,\,\,\,v = - \delta \frac{\partial \psi }{{\partial x}}.$$

where the following form represent the stress components:

15 $$S_{xx} = \frac{2\delta }{{\left( {1 + \lambda_{1} } \right)}}\left[ {1 + \frac{{\lambda_{2} c\delta }}{{d_{1} }}\left( {\psi_{y} \frac{\partial }{\partial x} - \psi_{x} \frac{\partial }{\partial y}} \right)} \right]\psi_{xy} ,$$

16 $$S_{xy} = \frac{1}{{\left( {1 + \lambda_{1} } \right)}}\left[ {1 + \frac{{\lambda_{2} c\delta }}{{d_{1} }}\left( {\psi_{y} \frac{\partial }{\partial x} - \psi_{x} \frac{\partial }{\partial y}} \right)} \right]\left( {\psi_{yy} - \delta^{2} \psi_{xx} } \right),$$

17 $$S_{yy} = \frac{2\delta }{{\left( {1 + \lambda_{1} } \right)}}\left[ {1 + \frac{{\lambda_{2} c\delta }}{{d_{1} }}\left( {\psi_{y} \frac{\partial }{\partial x} - \psi_{x} \frac{\partial }{\partial y}} \right)} \right]\psi_{xy} .$$

By adopting long wavelengths and low Reynolds numbers approximations in the previous nondimensional governing flow Eqs. (10)–(13),

18 $$0 = - \frac{dp}{{dx}} + \left( {\frac{1}{{1 + \lambda_{1} }}} \right)\frac{{\partial^{3} \psi }}{{\partial y^{3} }} + Gr\theta + Gm\Theta - \left( {M^{2} + \frac{1}{Da}} \right)\left[ {\frac{\partial \psi }{{\partial y}} + 1} \right] + \frac{{\text{Re}}}{Fr}\sin \eta ,$$

19 $$0 = - \frac{\partial p}{{\partial y}},$$

20 $$0 = \frac{{\partial^{2} \theta }}{{\partial y^{2} }} + Br\left[ {\left( {\frac{1}{{1 + \lambda_{1} }}} \right)\left( {\frac{{\partial^{2} \psi }}{{\partial y^{2} }}} \right)^{2} + M^{2} \left( {\frac{\partial \psi }{{\partial y}} + 1} \right)^{2} } \right] + \Pr Nb\frac{\partial \theta }{{\partial y}}\frac{\partial \Theta }{{\partial y}} + \Pr Nt\left( {\frac{\partial \theta }{{\partial y}}} \right)^{2} ,$$

21 $$0 = \frac{{\partial^{2} \Theta }}{{\partial y^{2} }} + \frac{Nt}{{Nb}}\frac{{\partial^{2} \theta }}{{\partial y^{2} }}.$$

The pressure gradient is eliminated in the obtained momentum Eqs. (18) and (19) via cross differentiation, the reconcilable equation can be obtained:

22 $$0 = \left( {\frac{1}{{1 + \lambda_{1} }}} \right)\frac{{\partial^{4} \psi }}{{\partial y^{4} }} + Gr\frac{\partial \theta }{{\partial y}} + Gm\frac{\partial \Theta }{{\partial y}} - \left( {M^{2} + \frac{1}{Da}} \right)\frac{{\partial^{2} \psi }}{{\partial y^{2} }}.$$

The following define the relevant governed boundary conditions:

23 $$\psi = \frac{q}{2},\frac{\partial \psi }{{\partial y}} = - 1,\,\,\theta = 0,\,\,\Theta = 0\,\,\,\,\,\,at\,\,\,\,y = + h = 1 + \varepsilon \sin \left( {2\pi x} \right),$$

24 $$\psi = - \frac{q}{2},\frac{\partial \psi }{{\partial y}} = - 1,\,\,\theta = 1,\,\,\Theta = 1\,\,\,\,\,at\,\,\,\,y = - h = - \left( {1 + \varepsilon \sin \left( {2\pi x} \right)} \right).$$

The flow rates in the fixed and wave frame and are connected by

25 $$Q = q + 1 + d.$$

The following equation defines the Nusselt and Sherwood numbers at the wall:

26 $$\,\,Nu = - \left. {\frac{\partial \theta }{{\partial y}}} \right|_{y = h} ,\,\,\,\,\,\,Sh = - \left. {\frac{\partial \Theta }{{\partial y}}} \right|_{y = h} .$$

## Numerical method

The Rung-Kutta method -Solve in MATLAB was used to numerically treat the transformed dimensionless equations. This approach demonstrates some advantages as it chooses the suitable algorithm and tracks automatically potential errors. Moreover, this method delivers enhanced computing results with minimal CPU time (3–4 min) per evaluation. Furthermore, it provides Graphical descriptions and eliminate intricate solution expressions. Nevertheless, it includes the Shooting technique that delivers graphical explanations using minimal to the maximal range. The Eqs. (20)–(22) subjected to boundary conditions (Eq. 23) and (Eq. 24) were numerically solved by the Rung-Kutta approach. The obtained results are validated with the solutions obtained by the bvp4c solver, a built-in function in the commercial software MATLAB. These governing Eqs. (20)–(22) of the nanofluid model are highly nonlinear and coupled. Getting the exact solution is challenging. Therefore, the numerical solution has been obtained. Moreover, excellent agreement is found in Table 1.

**Table 1:** Variations in physical quantities, Sherwood number and Nusselt number on the upper wall $h.$

$M$	$Gr$	$Nb$	$\lambda_{1}$	$\Pr$	$Da$	$Nu$	$Sh$
0.1	0.5	0.2	0.5	1	0.3	−0.689503	−0.223647
0.5						−0.777700	−0.135450
	1.0					−0.683573	−0.229576
	2.0					−0.672482	−0.240667
		1.0				−0.924424	−0.363005
		2.0				−1.276228	−0.374609
			1.0			−0.664026	−0.249123
			2.0			−0.637506	−0.275643
				1.5		−0.744003	−0.169147
				2		−0.801454	−0.111695
					0.7	−0.662973	−0.250177
					1.5	−0.650174	−0.262976

## Discussion

In the present work, the data available from our published papers are mostly new from this investigation.

The impact of relevant parameters on common profiles (concentration, temperature, and velocity) is discussed in this section. Additionally, the Sherwood numbers and numerical values of the reduced Nusselt are evaluated using the tabular results. The effects of Grashof number $Gr$, Hartmann number $M$, nanoparticle Grashof number $Gm,$ flow rates $Q,$ Darcy number $Da,$ Brinkman number $Br,$ Brownian motion parameter $Nb,$ thermophoresis parameter $Nt,$ the ratio of relaxation to retardation times $\lambda_{1}$ , and Prandtl number $\Pr$ on the peristaltic transport of a nanofluid are discussed in detail. The MATLAB inbuilt numerical Solver Rung-Kutta method was performed using the numerical computation.

**Figure 2:**
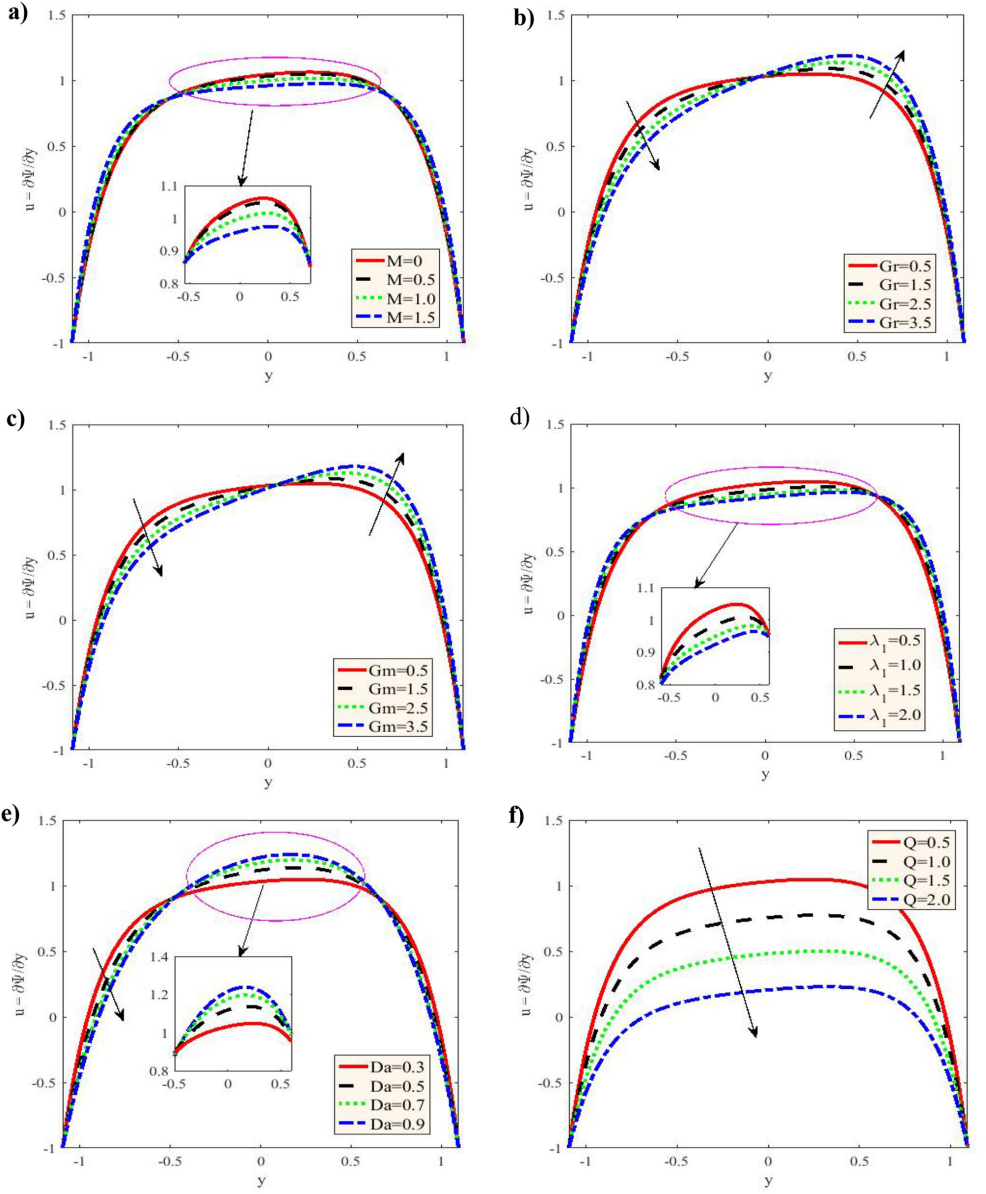
**(a)** Variation of $M$ in the velocity $u$ when $x = 0.3,\,\varepsilon = 0.1,\,Gr = 0.5,\,Gm = 0.5,\,\lambda_{1} = 0.5,$ $Br = 0.1,\,\Pr = 1,\,Nt = 0.2,\,Nb = 0.2,$ and $Da = 0.3.$ (**b)** Variation of $Gr$ on the velocity $u.$ **(c)** Variation of $Gm$ on the velocity $u.$ (**d)** Variation of $\lambda_{1}$ on the velocity $u.$ (**e)** Variation of $Da$ on the velocity $u.$ **(f)** Variation of $Q$ on the velocity $u.$

**Figure 3:**
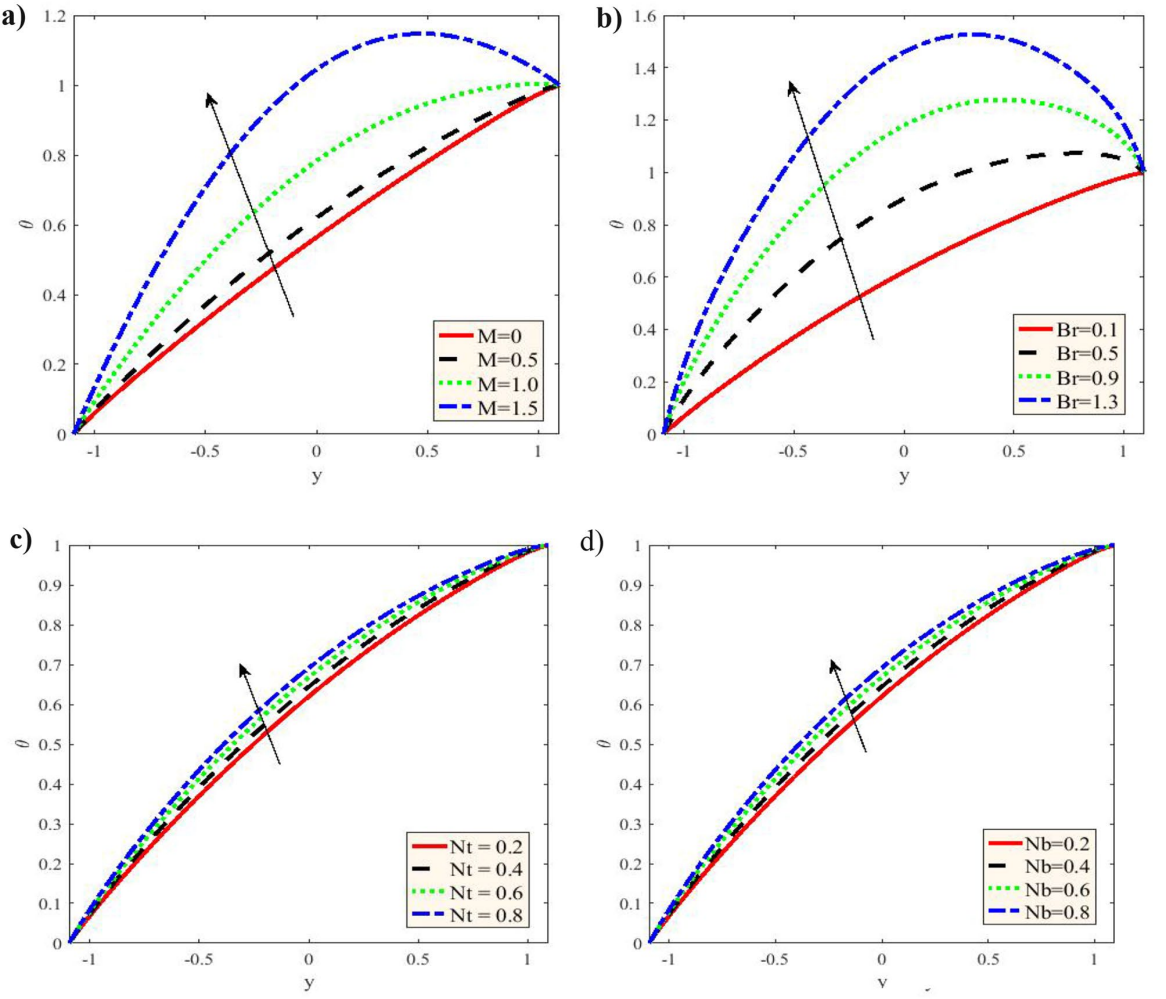
**(a)** Variation of $M$ in the temperature $\theta$ when $x = 0.3,\,\varepsilon = 0.1,\,Gr = 0.5,\,Gm = 0.5,\,\lambda_{1} = 0.5,$ $Br = 0.1,\,\Pr = 1,\,Nt = 0.2,\,Nb = 0.2,$ and $Da = 0.3.$ (**b)** Variation of $Br$ on the temperature $\theta .$ (**c)** Variation of $Nt$ on the temperature $\theta .$ **(d)** Variation of $Nb$ the temperature $\theta .$

Figure 2 is plotted for different values of the Grashof number $Gr,$ Hartmann number $M,$ nanoparticle Grashof number $Gm,$ ratio of relaxation to retardation times $\lambda_{1} ,$ Darcy number $Da$ and flow rates $Q.$ The velocity profiles are parabolic As shown in this figure. Additionally, with the increase of $M$ in the period $- 1.09 \le y \le - 0.5,$ the velocity elevates, and it decreases in the period $- 0.5 \le y \le 0.7,$ while it returns to rises in the period $0.7 \le y \le 1.09.$ For increasing $Gr,$ the velocity decreases within the period $- 1.09 \le y \le - 0.05,$ while it elevates in the period $- 0.05 \le y \le 1.09,$ nevertheless for increasing $Gm,$ the velocity decreases in the period $- 1.09 \le y \le 0.05,$;it increases in the period $0.05 \le y \le 1.09.$ Otherwise, with the increases in $\lambda_{1} ,$ cause the rise in the velocity in the period $- 1.09 \le y \le - 0.6,$ and it decreases in the interval $- 0.6 \le y \le 0.65,$ then it begin to rise in the period $0.65 \le y \le 1.09.$ In addition to, it has oscillatory and decreases with rising Darcy number in the period $- 1.09 \le y \le - 0.5,$ whereas it increases in the interval $- 0.5 \le y \le 0.7,$ while it decreases in the interval $0.7 \le y \le 1.09.$ Despite it decreases with increasing $Q$ in the whole range of $y - axis.$ A parabolic behavior is demonstrated by the current findings for velocity distribution curve, with its maximal value of occurs in the channel central part, while it decreases because of the impact of Hartman number. Moreover, the velocity profiles satisfy the boundary conditions. The temperature $\theta$ versus $y - axis$ is given in Fig. 3 to impact $M,\,Br,\,Nt\,$ and $Nb$, respectively. The temperature $\theta$ was observed to rise together with an increase in $M,\,Br,\,Nt$ and $Nb,$, which is consistent with the nanoparticles efficient movement from the wall to the fluid, leading to a significant rise in the temperature distribution. Additionally, this distribution is observed to satisfy the boundary conditions. The current findings are acquired numerical technique not as in ^19^ by an exact solution. The current approximate results demonstrated in Fig. 3 are consistent with Figs. 10 and 11 by Reddy and Makinde ^18^ with respect to the pertinent parameters.

**Figure 4:**
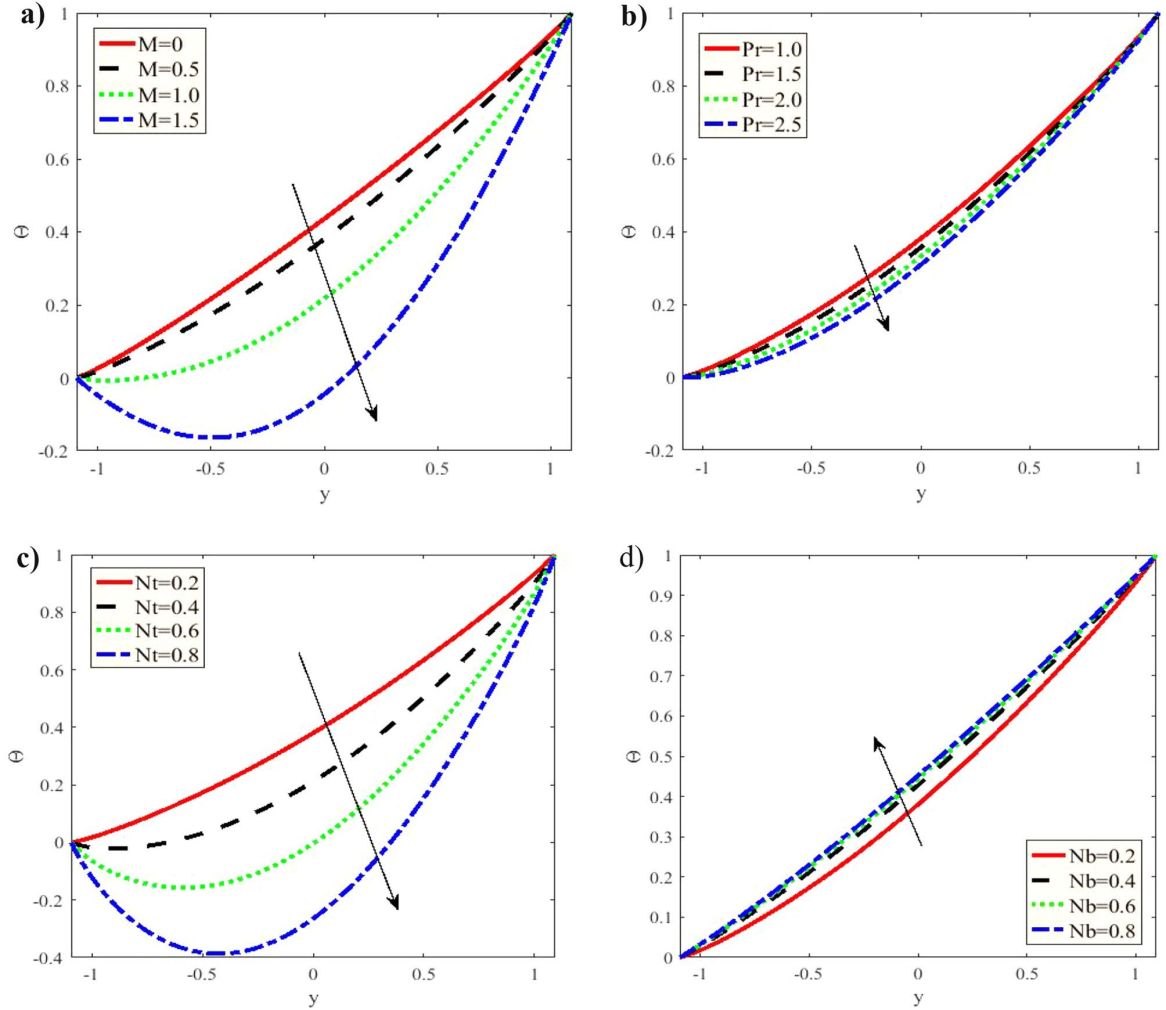
**(a)** Variation of $M$ in the concentration $\Theta$ when $x = 0.3,\,\varepsilon = 0.1,\,Gr = 0.5,\,Gm = 0.5,\,\lambda_{1} = 0.5,$ $Br = 0.1,\,\Pr = 1,\,Nt = 0.2,\,Nb = 0.2,$ and $Da = 0.3.$ (**b)** Variation of $\Pr$ on the concentration $\Theta .$ (**c)** Variation of $Nt$ on the concentration $\Theta .$ (**d)** Variation of $Nb$ the concentration $\Theta .$

Figure 4 demonstrates the varying concentration $$\Theta$$ against $$y - axis$$ for various values of $$M,$$$$\Pr ,$$$$Nt$$ and $$Nb.$$ The concentration is observed to decrease with rising $$M,$$$$\Pr ,$$$$Nt$$ while it increases with increasing $$Nb.$$ Nevertheless, the Brownian motion parameter effect on the concentration is insignificant. Furthermore, the concentration distribution seems to satisfy the boundary conditions. The $$Nt$$ smaller values significantly influence the concentration function. Figure 4 demonstrates that the concentration function magnitude dramatically lessens with a rise in $$Nt$$.This is consistent with what was acquired in clinical practice as the nutrients diffuse out of the blood vessels to neighboring tissues ^46^.

The discrepancy in reduced Sherwood and reduced Nusselt numbers regarding pertinent parameters different values is shown in Table 1. The increasing values are observed in Hartmann number $M$, Grashof number $Gr$, Darcy number $Da,$ Brownian motion parameter $Nb,$ Prandtl number $\Pr$ and the ratio of relaxation to retardation times $\lambda_{1}$ decline and increase the heat and mass transfer rate.

## Conclusion

The Jeffrey nanofluid peristaltic flow within an inclined symmetric channel was studied because of the critical applications in medicine, chemistry, and biomedical engineering. The exact solution is obtained for concentration, velocity, temperature. Furthermore, the Jeffrey nanofluid model governing equations was obtained. The flow nondimensional governing equations were solved numerically utilizing the Rung-Kutta approach. This study findings were as follows:

I. The velocity decreases in the middle of the channel, while the inverse behavior diminishes in the walls of the channel.
II. The fluid velocity profile represents an increasing function close to the upper channel for Gr and Gm.
III. The velocity distribution experienced a reverse trend on the walls of the channel to the Jeffrey nanofluid Darcy number $Da.$
IV. An increase in $Nb,$ $Nt,$ $Br$ and $M.$, increases The temperature profile.
V. It can be concluded that the Newtonian fluid peristaltic flow under the magnetic field influence is higher than that of nanofluid under the magnetic field impact (Fig. 2).
VI. The behavior of concentration and temperature are present and it has fulfilled the boundary conditions.

### Future perspectives

The Lobatto IIIA scheme may be implemented for the numerical treatment of various prospective applications appearing in bioinformatics, fluid mechanics problems, financial mathematics of vital significance^28–47^.

## Abbreviations

$$a$$: The amplitude, m
$\mu$: The viscosity constant of the nanofluid dynamics, kg m^−^^1^ s^−^^1^
$2d$: Width of the channel, m
$$\lambda$$: Wavelength, m
$$c$$: Wave speed, m s^−^^1^
$\overline{U} ,\overline{V}$: Velocity components in the fixed frame, m s^−^^1^
$\eta$: The inclination of the symmetric channel to the vertical
$\overline{{\mathop \gamma \limits^{.} }}$: The shear rate
$\rho_{f}$: Nanofluid’s density, kg m^−^^3^
$\rho_{p}$: The density of the fluid at a pressure constant
$\rho_{c}$: The density of the fluid at constant velocity
$\lambda_{1}$: The ratio of relaxation to retardation times
$\lambda_{2}$: The retardation time
$\overline{t}$: Time in the fixed frame, s
$g$: Increasing speed due to gravity, m s^−^^2^
$\overline{P}$: Pressure in the fixed frame, kg m^−^^1^ s^−^^2^
$$k_{1}$$: The permeability, m^2^
$\sigma$: Electrical conductivity of the fluid, S m^−^^1^
$\beta_{1}$: Coefficient of thermal expansion, K^−^^1^
$\beta_{2}$: Coefficient of viscosity at constant concentration, K^−^^1^
$T_{1} ,T_{o}$: The walls temperature
$C_{1} ,C_{o}$: The walls concentration
$T$: The temperature of the fluid, K
$C$: The concentration of the fluid, mol m^−^^3^
$B_{0}$: The intensity of the external magnetic field, Tesla
$\alpha$: The thermal diffusivity, m^2^ s^−^^1^
$\tau$: The effective heat capacity
$c_{f}$: Specific heat of the nanofluid, J kg^−^^1^ K^−^^1^
$D_{B}$: Brownian motion coefficient
$D_{T}$: Thermophoretic diffusion coefficient
$T_{m}$: The mean temperature
$\upsilon$: The kinematic viscosity, m^2^ s^−^^1^
$c_{p}$: Specific heat, J kg^−^^1^ K^−^^1^
$\varepsilon$: The wave amplitude
$$\theta$$: Temperature distribution
$$\Theta$$: Concentration distribution
$$\delta$$: Wavenumber
$$\Pr$$: Prandtl number
$${\text{Re}}$$: Reynolds number
$$M$$: Hartmann number
$$Gr$$: Grashof number
$Gm$: Nanoparticle Grashof number
$$Br$$: Brinkman number
$$Nt$$: Thermophoresis parameter
$$Ec$$: Eckert number
$$Da$$: Darcy number
*Nb*: Brownian motion parameter
$\Psi$: The stream function
*q*: The flux of the nanofluid
*Fr*: Froude number
